# C-reactive protein flare-response predicts the efficacy of PD-1 inhibitors in metastatic gastric cancer

**DOI:** 10.3389/fimmu.2026.1802864

**Published:** 2026-07-01

**Authors:** Yi-hui Lei, Hui Zheng, Xin-fu Song, Ya-yue Wang, Feng-bin Cai

**Affiliations:** 1 Department of Gastrointestinal Surgery, Xiamen Humanity Hospital, Xiamen, Fujian, China; 2 School of Nursing, Fujian Medical University, Fuzhou, Fujian, China; 3 School of Medicine, Xiamen University, Xiamen, Fujian, China

**Keywords:** early C-reactive protein kinetics, immunotherapy, metastatic gastric cancer, microsatellite stability, programmed cell death protein-1

## Abstract

**Background:**

Currently, predictive biomarkers for the efficacy of immunotherapy in metastatic gastric cancer (mGC) during the era of immune checkpoint inhibitors are still under evaluation. The aim of this study is to investigate the predictive value of early C-reactive protein (CRP) kinetics on the efficacy of programmed cell death protein-1(PD-1) inhibitors in mGC patients with microsatellite stable (MSS).

**Methods:**

This retrospective study included 59 mGC patients with MSS who had been treated with PD-1 inhibitors as first-line therapy at a tertiary hospital. Based on the “CRP-flare response phenomenon,” the patients were classified into three groups: CRP flare-responders, CRP responders, and non-CRP responders. The primary endpoints were overall survival (OS) and progression-free survival (PFS), while the secondary endpoints included objective response rate.

**Results:**

Among the 59 patients, 21 were classified as CRP flare-responders, 18 as CRP responders, and 20 as non-CRP responders. The objective response rate for CRP flare-responders, CRP responders, and non-CRP responders were 38.1%, 16.7%, and 5.0% (p=0.031), respectively. The median OS of the CRP flare-responders, CRP responders, and non-CRP responders were 25.8 months, 16.3 months, and 11.9 months (p=0.0009), respectively. The median PFS of the CRP flare-responders, CRP responders, and non-CRP responders were 16.6 months, 6.8 months, and 5.1 months (p=0.0002), respectively. Both univariable and multivariable analyses demonstrated that CRP flare-responders had a significantly higher objective response rate and a lower risk of death compared with non-CRP responders (p < 0.05). Moreover, CRP flare-responders showed a significantly lower risk of disease progression compared with both CRP responders and non-CRP responders (p < 0.05).

**Conclusions:**

Among patients with MSS mGC receiving first-line PD-1 inhibitor therapy, CRP flare response was associated with improved objective response, PFS, and OS. Early CRP kinetics may represent a promising predictive biomarker for immunotherapy efficacy in MSS mGC. Further large-scale prospective studies are warranted to validate these findings.

## Introduction

In recent years, various novel immune checkpoint inhibitors have emerged, and programmed cell death protein-1(PD-1) blockade-based immunotherapies have shown potential to improve survival outcomes in patients with metastatic gastric cancer (mGC) (1–3). However, not all mGC patients respond to PD-1 inhibitors therapy. The efficacy of immunotherapy is influenced by a multitude of factors, including tumor molecular characteristics, host-related factors, and the tumor microenvironment. Consequently, careful patient selection—such as identifying individuals with high programmed death-ligand 1 expression—and providing psychological interventions for patients experiencing emotional distress may contribute to improved therapeutic outcomes (4, 5). Nevertheless, the majority of patients with gastric cancer exhibit microsatellite stability (MSS), which is generally associated with limited responsiveness to immunotherapy (6, 7). Therefore, there is an urgent need for reliable predictive biomarkers to identify early treatment efficacy, enabling timely adjustments to individualized treatment plans.

The antitumor efficacy of PD-1 inhibitors is typically associated with the activation of anti-tumor immune responses (8). The immune responses are closely associated with the inflammation. Cytokines secreted by immune cells play crucial roles in stimulating, inducing, and regulating inflammation (9).Therefore, serum inflammatory markers hold significant value in predicting antitumor immune activation.

Generally, chronic inflammation, through factors produced within immune cells, leads to the creation of an inhibitory tumor immune microenvironment. This diminishes the efficacy of anticancer therapies, thereby promoting tumor invasion and growth (10). However, the impact of acute inflammatory responses on the treatment outcomes of solid tumors remains incompletely understood.

C-reactive protein (CRP), as a readily obtainable marker of systemic acute inflammation, is widely used clinically to assess the severity of systemic inflammation. Previously, we reported individual cases evaluating the predictive value of early CRP level changes for the efficacy of PD-1 inhibitors treatment in advanced gastrointestinal tumors (11). Furthermore, Fukuda et al. (12) introduced the concept of the “CRP-flare response phenomenon” and established the classification criteria for early CRP kinetics. This has been shown to be associated with the therapeutic efficacy of immune checkpoint inhibitors across various solid tumors (12–14).

However, the predictive value of early CRP kinetics in patients with MSS mGC receiving PD-1 inhibitor-based therapy remains unclear. Therefore, we conducted this study to evaluate the association between early CRP kinetics and treatment outcomes and to explore its potential as a predictive biomarker for immunotherapy efficacy in this population.

## Materials and methods

### Study design and patients

This retrospective cohort study was conducted at a tertiary medical institution and included patients with MSS mGC who received PD-1 inhibitors as first-line therapy. Patient data was collected from January 2018 to September 2022. The inclusion criteria were as follows (1): confirmed mGC through endoscopy, pathological diagnosis, magnetic resonance imaging, or computed tomography scans; (2) genetic testing confirmed MSS status; (3) receipt of PD-1 inhibitors as first-line therapy, with or without concomitant treatments; (4) availability of complete clinical and follow-up data; (5) PD-1 inhibitors were administered every 2 weeks or 3 weeks until radiographic or clinical disease progression, death, or the occurrence of intolerable adverse events. The exclusion criteria were as follows: (1) history or presence of other primary malignancies; (2) patients with immune-related diseases; (3) Eastern Cooperative Oncology Group performance status (ECOG PS)>2; (4) history of bone marrow suppression.

### Data collection and outcome

Demographic and clinical characteristics included: age, sex, ECOG PS, degree of tumor differentiation, history of gastrectomy, metastatic sites, HER2 status, combination therapy regimen, and baseline CRP levels. Patient survival, tumor progression, and response were collected and recorded through telephone follow-ups, outpatient records, and inpatient records. Serum CRP levels were measured 3 to 7 days before the initiation of PD-1 inhibitors therapy, and subsequently at least every 2 weeks during treatment. All CRP data in this study were collected by investigators who were not involved in CRP response classification or survival analyses. Imaging assessments, including computed tomography scans, magnetic resonance imaging, and/or bone scans, were repeated approximately every 8 to 12 weeks to evaluate disease status and progression. Treatment was administered according to the approved dosing schedules of the respective regimens until radiographic or clinical disease progression, death, or intolerable adverse events occurred. The primary endpoints were overall survival (OS) and progression-free survival (PFS), while the secondary endpoint was objective response rate (ORR). The assessment of objective response (OR) was based on the Response Evaluation Criteria in Solid Tumors version 1.1.

### Classification of patients

Patients were categorized into three groups: (1) CRP flare-responders, defined as patients whose CRP levels increased to more than twice the baseline value and exceed 10 mg/L within six weeks after initiation of PD-1 inhibitor therapy, followed by a decline below baseline level within three months; (2) CRP responders, defined as patients with baseline CRP levels >10 mg/L whose CRP levels decrease by more than 30% within three months after treatment initiation; (3) non-CRP responders: the remaining patients. CRP levels measured before both the first and second treatment cycles were considered baseline values.

### Statistical analysis

Categorical variables were compared using the Pearson’s chi-square test, or Fisher’s exact test. Numerical variables were compared using ANOVA or Kruskal–Wallis rank-sum test. Firth-penalized logistic regression was employed to identify predictors of OR. Schoenfeld residual testing was performed to assess whether the relevant variables satisfied the proportional hazards assumption. PFS and OS were estimated using Kaplan–Meier survival analysis, and group differences were assessed using the log-rank test. Firth-penalized Cox proportional hazards regression analysis was performed to evaluate factors associated with survival outcomes. Variables with a p value ≤ 0.20 in the univariable analysis, metastasis-related variables meeting the criteria of a baseline p value ≤ 0.20 and n > 15, and clinically relevant factors considered likely to affect treatment outcomes were entered into the multivariable model. *Post hoc* power analysis and sensitivity analyses were performed to evaluate the reliability and robustness of the survival outcomes. In addition, propensity score matching (PSM) was performed to reduce potential confounding. Variables potentially associated with survival outcomes in the Cox regression analysis were selected as matching covariates. A 1:1 nearest-neighbor matching algorithm with a caliper width of 0.02 was applied, and survival analyses were subsequently conducted in the matched cohort. All analyses were two-sided, and differences were considered significant at p<0.05. All statistical analyses were performed using RStudio version 4.5.1 and GPower version 3.1.9.7, and graphs were created using Origin version 2022 and RStudio version 4.5.1.

## Result

### Patient’s characteristics at baseline

A total of 59 mGC patients were included, with baseline characteristics shown in **Table 1**. The median age of the patients was 63 years. There were 36 male patients (61%) and 23 female patients (39%). 49 patients (83%) had an ECOG PS of 0-1. 49 patients (83%) had poorly differentiated adenocarcinoma. HER-2 status was negative in 51 patients (86%). 23 patients (39%) had undergone palliative gastrectomy. Among all patients, 3 patients (5%) received PD-1 inhibitor monotherapy, 43 patients (73%) received PD-1 inhibitors combined with chemotherapy, 1 patient (2%) received PD-1 inhibitors combined with targeted therapy, 12 patients (20%) received PD-1 inhibitors combined with chemotherapy and targeted therapy. The median CRP level before the first treatment was 7.7 mg/L (IQR, 5.4-16.7). Within the current sample size, no significant differences were observed among the three groups with respect to the baseline characteristics presented in **Table 1**.

**Table 1:** Patient characteristics.

Variables		Total cohort, n (%)	CRP flare-responder, n (%)	CRP responder, n (%)	Non-CRP responder, n (%)	P value
No. of patients		59	21(36)	18(30)	20(34)	
Age (years)	Median (IQR)	63(59-68)	65(60-69)	63(60-67)	60(55-63)	0.291
Gender	Male	36(61)	14(67)	9(50)	13(65)	0.513
	Female	23(39)	7(33)	9(50)	7(35)	
ECOG PS	0	25(42)	9(43)	10(56)	6(30)	0.591
	1	24(41)	9(43)	5(28)	10(50)	
	2	10(17)	3(14)	3(17)	4(20)	
Differentiation	Poor	49(83)	16(76)	14(78)	19(95)	0.231
	Moderate	10(17)	5(24)	4(22)	1(5)	
HER-2	Positive	8(14)	5(24)	2(11)	1(5)	0.220
	Negative	51(86)	16(76)	16(89)	19(95)	
Gastrectomy	Yes	23(39)	10(48)	5(28)	8(40)	0.445
	No	36(61)	11(52)	13(72)	12(60)	
Sites of metastases	Lung	9(15)	3(14)	3(17)	3(15)	1.000
	Bone	6(10)	0(0)	3(17)	3(15)	0.126
	Peritoneum	25(42)	12(57)	7(39)	6(30)	0.200
	lymph node	10(17)	4(19)	2(11)	4(20)	0.757
	Liver	18(31)	5(24)	9(50)	4(20)	0.124
	Ovary	4(7)	0(0)	1(6)	3(15)	0.140
	Kidney	1(2)	1(5)	0(0)	0(0)	1.000
Combination therapy	No	3(5)	2(10)	0(0)	1(5)	0.842
	Chemo	43(73)	14(67)	15(83)	14(70)	
	Targeted	1(2)	0(0)	0(0)	1(5)	
	Chemo + targeted	12(20)	5(24)	3(17)	4(20)	
CRP level before the first treatment (mg/L)	Median (IQR)	7.7(5.4-16.7)	6.8(5.0-11.0)	13.8 (8.6-24.8)	6.0(2.7-13.0)	0.141

ECOG PS, Eastern Cooperative Oncology Group performance status; HER-2, human epidermal growth factor-2; chemo, chemotherapy; targeted, targeted therapy.

**Figure 1:**
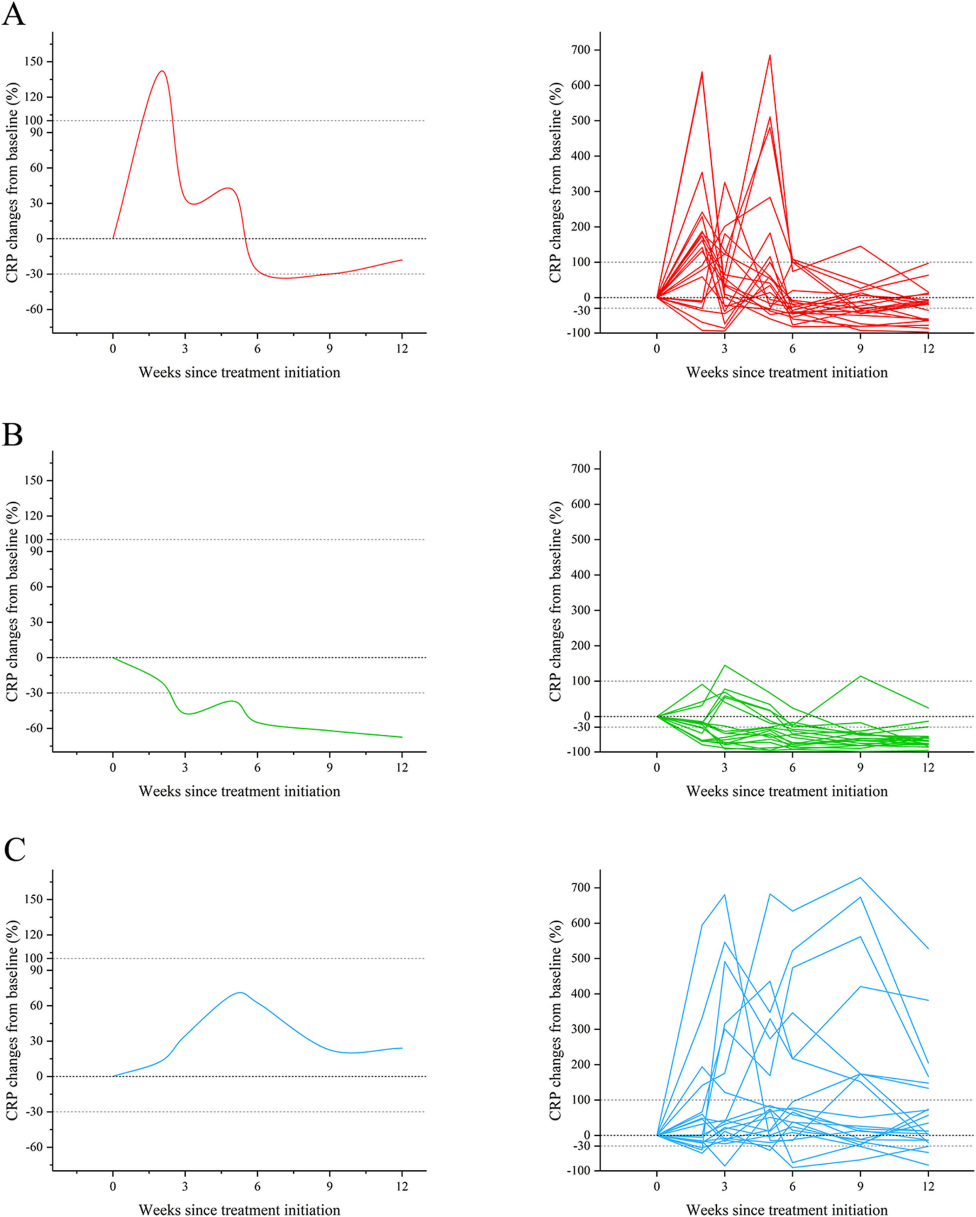
CRP changes from baseline after initiation of immunotherapy. **(A)** for CRP flare-responders, **(B)** for CRP responders, and **(C)** for CRP non-responders: median CRP change from baseline in per cent is shown in the left panel; the CRP changes of the individual patients are shown in the right panel.

The changes in CRP levels of the patients are depicted in **Figure 1**. In the CRP flare-responders, 19 patients exhibited a CRP level that doubled within six weeks compared to first pre-treatment level. Among CRP responders, 12 patients with baseline CRP levels ≥10 mg/L experienced a decrease of more than 30% within three months after treatment initiation.

### Tumor shrinkage rate and objective response by early CRP kinetics

**Figure 2:**
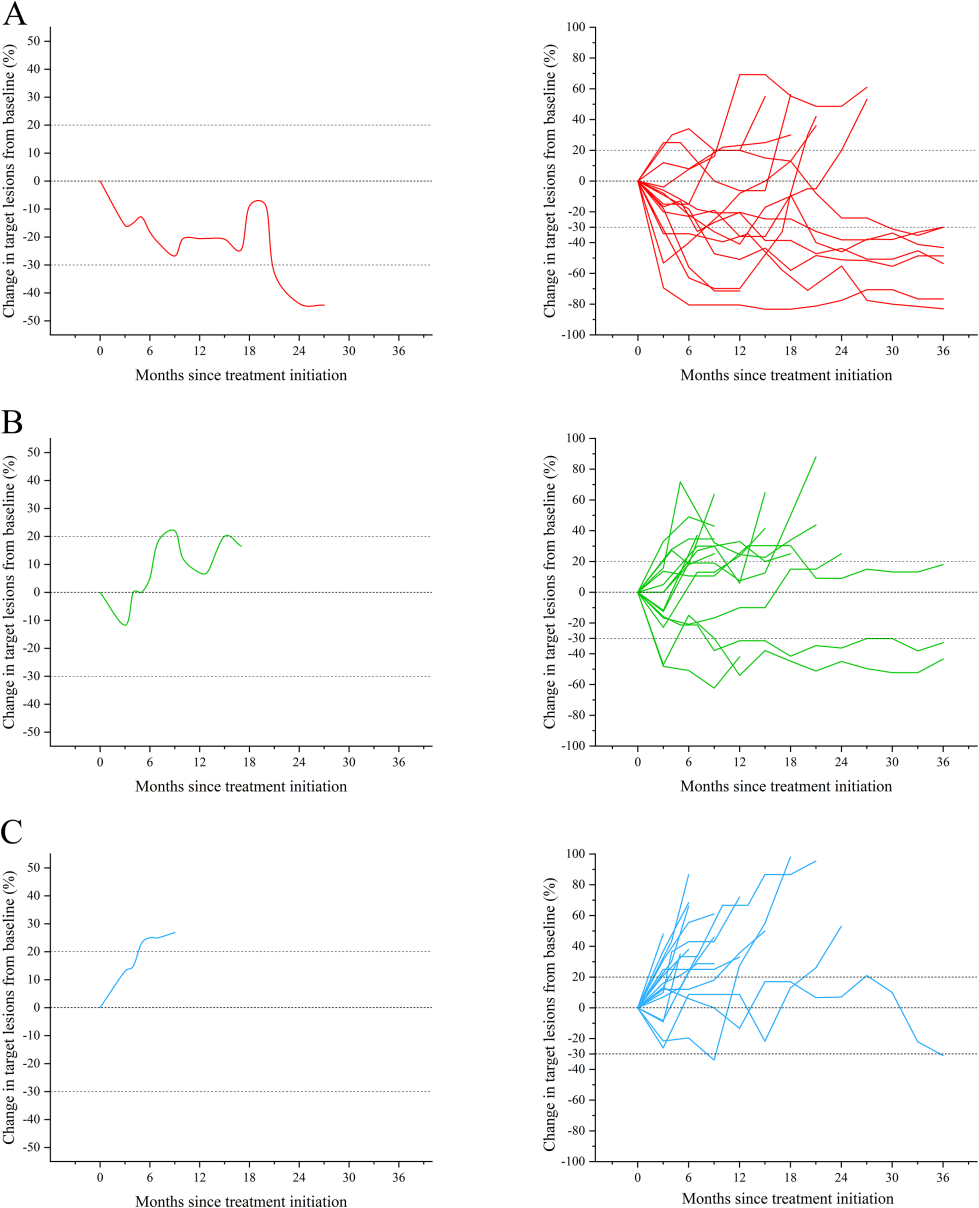
Change in target lesions from baseline based on early CRP kinetics. **(A)** for CRP flare-responders, **(B)** for CRP responders, and **(C)** for CRP non-responders: median target lesions change from baseline in per cent is shown in the left panel; the target lesions changes of the individual patients are shown in the right panel. Each group will cease recording the overall percentage change in target lesions once the number of follow-up participants reaches half of the initial count, though the percentage change for individual target lesions will continue to be recorded.

The changes in the target lesions of the patients are illustrated in **Figure 2**. At the end of the follow-up period, 12 patients (20.3%) achieved partial response, 2 patients (3.4%) had stable disease, and 45 patients (76.3%) experienced disease progression. In the CRP flare-responders, CRP responders, and non-CRP responders, 8, 3, and 1 patient, respectively, achieved OR (p = 0.031). In both univariable and multivariable analyses of OR, CRP flare-responders were significantly more likely to achieve OR than non-CRP responders (all p < 0.05) (**Table 2**). However, no significant difference was observed between CRP flare-responders and CRP responders (all p > 0.05) (**Supplementary Table 1**).

**Table 2:** Univariate and multivariate analysis for objective response.

Univariate analysis				
		OR	95%CI	P value
Group	CRP flare-responder	8.19	1.56 to 83.52	0.011
	CRP responder	2.94	0.43 to 32.66	0.277
	Non-CRP responder	Reference	Reference	Reference
Age	≥65	1.91	0.54 to 6.75	0.308
	<65	Reference	Reference	Reference
Gender	Male	0.86	0.25 to 3.10	0.806
	Female	Reference	Reference	Reference
ECOG PS	0	1.70	0.28 to 18.27	0.584
	1	2.23	0.38 to 23.63	0.395
	2	Reference	Reference	Reference
Differentiation	Poor	1.89	0.37 to 18.95	0.474
	Moderate	Reference	Reference	Reference
HER-2	Positive	1.52	0.25 to 7.07	0.617
	Negative	Reference	Reference	Reference
Gastrectomy	Yes	1.17	0.32 to 4.05	0.806
	No	Reference	Reference	Reference
Combination therapy	Chemo	1.16	0.27 to 6.77	0.853
	Chemo + targeted	Reference	Reference	Reference
Liver metastasis	Yes	1.22	0.31 to 4.37	0.762
	No	Reference	Reference	Reference
Peritoneum metastasis	Yes	0.65	0.17 to 2.27	0.507
	No	Reference	Reference	Reference
CRP level before the first treatment (mg/L)	≥10	0.45	0.10 to 1.64	0.236
	<10	Reference	Reference	Reference
Multivariate analysis				
		OR	95%CI	P value
Model 1	CRP flare-responder	7.91	1.53 to 80.11	0.012
Model 2	CRP flare-responder	7.89	1.52 to 79.88	0.012
Model 3	CRP flare-responder	11.01	1.94 to 120.34	0.005
Model 4	CRP flare-responder	7.24	1.41 to 73.29	0.016

ECOG PS, Eastern Cooperative Oncology Group performance status; HER-2, human epidermal growth factor-2; chemo, chemotherapy; targeted, targeted therapy.

Model 1: include the group variable and the gastrectomy variable.

Model 2: include the group variable and the liver metastasis variable.

Model 3: include the group variable and the peritoneum metastasis variable.

Model 4: include the group variable and the combination therapy variable.

### Survival analysis by early CRP kinetics

**Figure 3:**
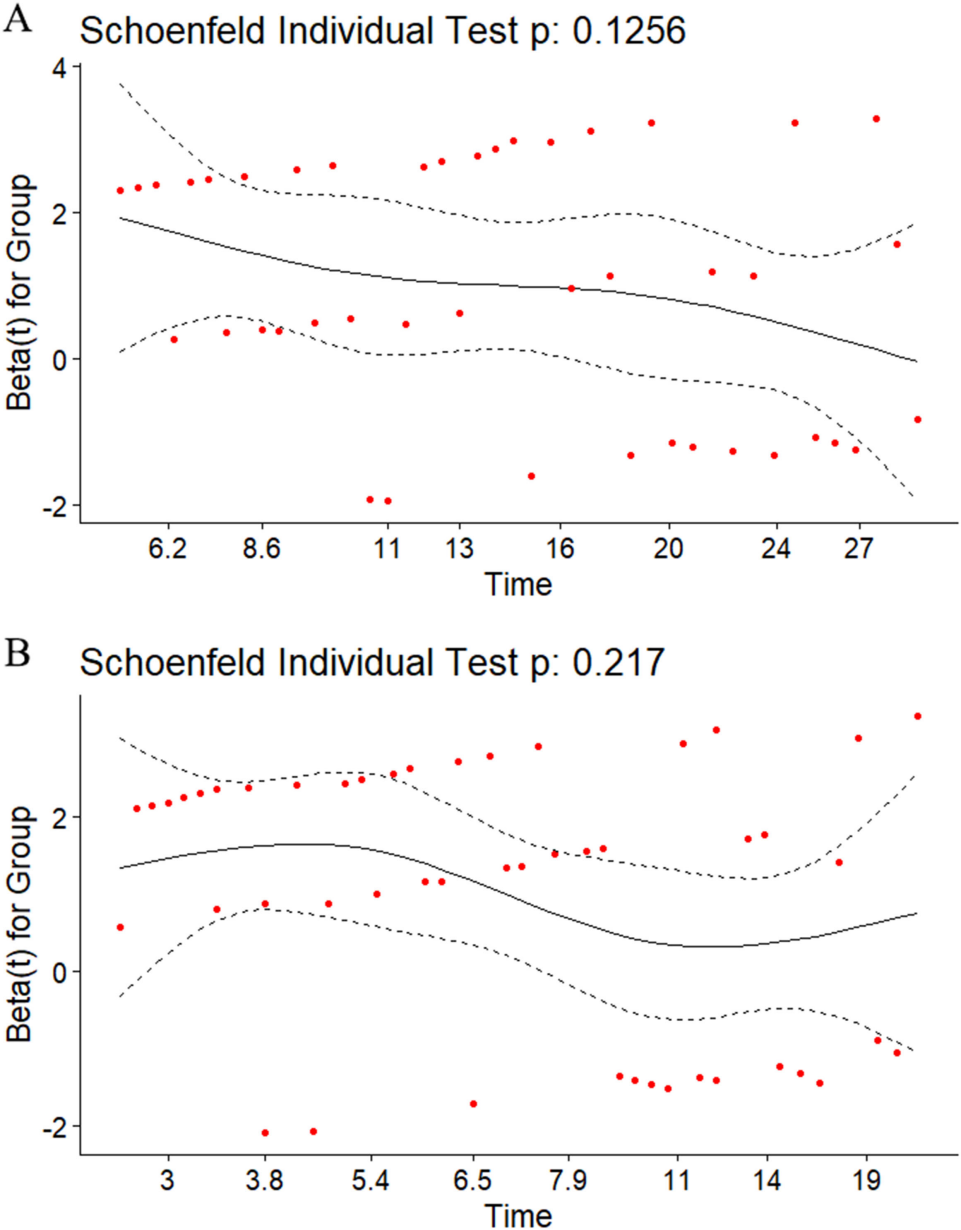
Schoenfeld residual analysis for **(A)** overall survival and for **(B)** progression-free survival across the three groups.

Schoenfeld residual testing confirmed that the group variable did not violate the proportional hazards assumption in the log-rank analyses of OS and PFS, with all p-values exceeding 0.05 (**Figure 3**).

**Figure 4:**
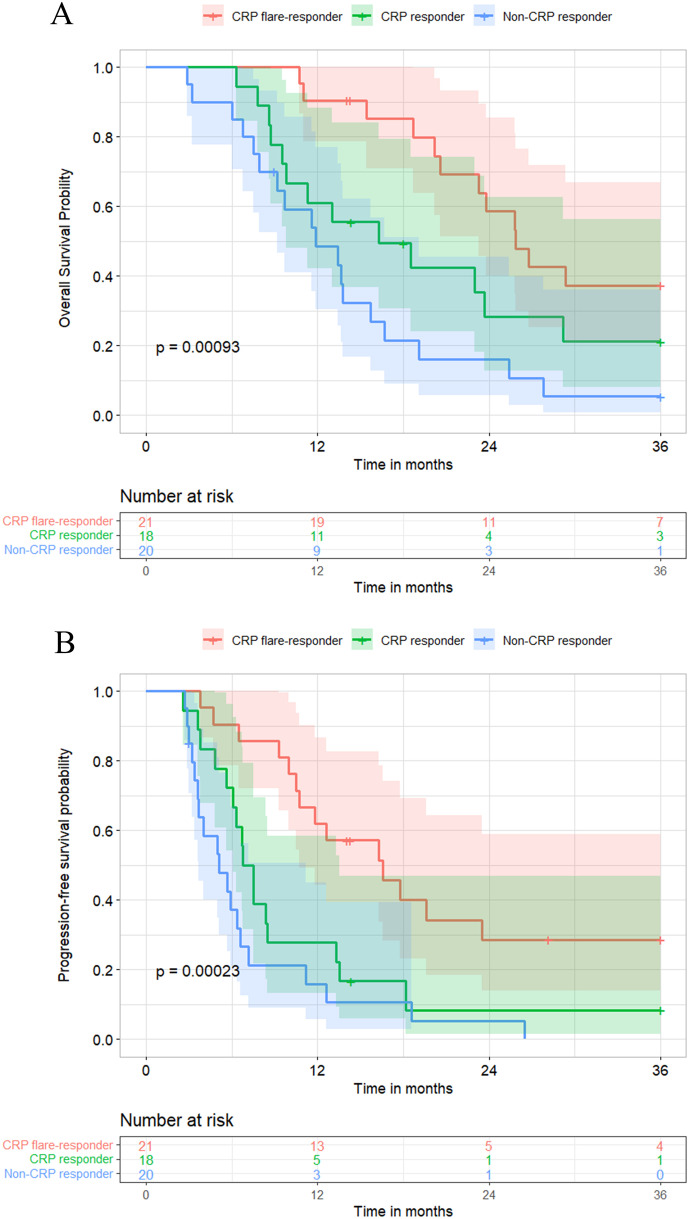
**(A)** overall survival curves and **(B)** progression-free survival curves based on early CRP kinetics.

The median follow-up time for the entire cohort was 36 months. Five patients were lost to follow-up, including two CRP flare-responders, two CRP responders, and one non-CRP responder. The OS curve for the entire cohort is shown in **Figure 4A**. The median OS of the CRP flare-responders, CRP responders, and non-CRP responders were 25.8 months (95% CI, 21.6-30.1 months), 16.3 months (95% CI, 6.5-26.1 months), and 11.9 months (95% CI, 6.7-17.1 months), respectively, with significant differences observed among the three groups (p=0.00093). *Post hoc* power analysis demonstrated that, with a significance level of α = 0.05 and a target statistical power of 0.95, the achieved power was 0.9578.

The PFS curve for the entire cohort is illustrated in **Figure 4B**. The median PFS of the CRP flare-responders, CRP responders, and non-CRP responders were 16.6 months (95% CI, 9.9-23.3 months), 6.8 months (95% CI, 5.7-7.9 months), and 5.1 months (95% CI, 2.8-7.4 months), respectively, with significant differences observed among the three groups (p=0.00023). *Post hoc* power analysis demonstrated that, with a significance level of α = 0.05 and a target power of 0.95, the achieved statistical power was 0.9534.

### Cox regression analysis

Schoenfeld residual tests confirmed that the proportional hazards assumption was not violated for any covariates included in the multivariable models or for the global tests of the OS and PFS models (all p > 0.05) (**Supplementary Figures 1**, **2**).

In the univariable analysis of OS and PFS, a significant survival difference was observed only for the group variable. Compared with CRP non-responders, CRP flare-responders exhibited a significantly lower risk of death (p = 0.0003, HR = 0.26, 95% CI: 0.12–0.54) and lower risk of progression (p = 0.0001, HR = 0.25, 95% CI: 0.12–0.50) (**Tables 3**, **4**). Compared with CRP responders, CRP flare-responders showed a trend toward a lower risk of death, although this was not statistically significant (p = 0.073, HR = 0.49, 95% CI: 0.22–1.07). In contrast, the risk of disease progression was significantly reduced (p = 0.014, HR = 0.40, 95% CI: 0.19–0.83) (**Supplementary Tables 2**, **3**).

**Table 3:** Univariate and multivariate analysis for overall survival.

Univariate analysis				
	Variable	HR	95%CI	P value
Group	CRP flare-responder	0.26	0.12 to 0.54	0.0003
	CRP responder	0.54	0.26 to 1.08	0.0839
	Non-CRP responder	Reference	Reference	Reference
Age	≥65	0.80	0.41 to 1.47	0.4739
	<65	Reference	Reference	Reference
Gender	Male	1.09	0.60 to 2.05	0.7834
	Female	Reference	Reference	Reference
ECOG PS	0	0.45	0.20 to 1.03	0.0593
	1	0.62	0.29 to 1.43	0.2525
	2	Reference	Reference	Reference
Differentiation	Poor	0.92	0.45 to 2.10	0.8273
	Moderate	Reference	Reference	Reference
HER-2	Positive	0.79	0.33 to 1.62	0.5358
	Negative	Reference	Reference	Reference
Gastrectomy	Yes	0.91	0.48 to 1.66	0.7613
	No	Reference	Reference	Reference
Combination therapy	Chemo	1.72	0.85 to 3.93	0.1371
	Chemo + targeted	Reference	Reference	Reference
Liver metastasis	Yes	1.03	0.52 to 1.91	0.9389
	No	Reference	Reference	Reference
Peritoneum metastasis	Yes	1.17	0.64 to 2.12	0.6119
	No	Reference	Reference	Reference
Baseline CRP level (mg/L)	≥10	1.36	0.74 to 2.46	0.3159
	<10	Reference	Reference	Reference
Multivariate analysis				
	Variable	HR	95%CI	P value
Model 1	CRP flare-responder	0.26	0.12 to 0.54	0.0003
	Gastrectomy (yes)	1.10	0.57 to 2.04	0.7714
Model 2	CRP flare-responder	0.25	0.11 to 0.53	0.0003
	Liver metastasis (yes)	1.26	0.58 to 2.67	0.5505
Model 3	CRP flare-responder	0.26	0.12 to 0.53	0.0003
	Peritoneum metastasis (yes)	1.31	0.71 to 2.37	0.3889
Model 4	CRP flare-responder	0.23	0.11 to 0.51	0.0002
	Chemotherapy	2.15	1.04 to 5.00	0.0391
Model 5	CRP flare-responder	0.23	0.10 to 0.49	0.0001
	ECOG PS (0)	0.35	0.16 to 0.84	0.0195
Model 6	CRP flare-responder	0.27	0.12to 0.56	0.0005
	HER-2 (positive)	0.41	0.13 to 0.99	0.0478

ECOG PS, Eastern Cooperative Oncology Group performance status; HER-2, human epidermal growth factor-2; chemo, chemotherapy; targeted, targeted therapy.

Model 1: include the group variable and the gastrectomy variable.

Model 2: include the group variable and the liver metastasis variable.

Model 3: include the group variable and the peritoneum metastasis variable.

Model 4: include the group variable and the combination therapy variable.

Model 5: include the group variable and the ECOG PS variable.

Model 6: include the group variable and the HER-2 variable.

**Table 4:** Univariate and multivariate analysis for progression-free survival.

Univariate analysis				
	Variable	HR	95%CI	P value
Group	CRP flare-responder	0.25	0.12 to 0.50	0.0001
	CRP responder	0.62	0.32 to 1.21	0.1613
	Non-CRP responder	Reference	Reference	Reference
Age	≥65	0.69	0.37 to 1.22	0.2024
	<65	Reference	Reference	Reference
Gender	Male	0.87	0.50 to 1.54	0.6255
	Female	Reference	Reference	Reference
ECOG PS	0	0.50	0.24 to 1.09	0.0800
	1	0.53	0.26 to 1.17	0.1142
	2	Reference	Reference	Reference
Differentiation	Poor	0.80	0.41 to 1.76	0.5633
	Moderate	Reference	Reference	Reference
HER-2	Positive	0.79	0.33 to 1.62	0.5358
	Negative	Reference	Reference	Reference
Gastrectomy	Yes	0.92	0.51 to 1.62	0.7711
	No	Reference	Reference	Reference
Combination therapy	Chemo	1.62	0.84 to 3.41	0.1538
	Chemo + targeted	Reference	Reference	Reference
Liver metastasis	Yes	1.06	0.56 to 1.92	0.8508
	No	Reference	Reference	Reference
Peritoneum metastasis	Yes	0.92	0.52 to 1.61	0.7689
	No	Reference	Reference	Reference
CRP level before the first treatment (mg/L)	≥10	1.25	0.71 to 2.18	0.4366
	<10	Reference	Reference	Reference
Multivariate analysis				
	Variable	HR	95%CI	P value
Model 1	CRP flare-responder	0.23	0.11 to 0.47	0.00008
	Gastrectomy (yes)	1.32	0.71 to 2.40	0.3782
Model 2	CRP flare-responder	0.25	0.12 to 0.50	0.0001
	Liver metastasis (yes)	1.10	0.55 to 2.11	0.7793
Model 3	CRP flare-responder	0.24	0.11 to 0.48	0.00009
	Peritoneum metastasis (yes)	1.24	0.68 to 2.23	0.4738
Model 4	CRP flare-responder	0.19	0.08 to 0.41	0.0002
	Chemotherapy	2.59	1.23 to 5.93	0.0108
Model 5	CRP flare-responder	0.23	0.10 to 0.49	0.00005
	ECOG PS (0)	0.41	0.19 to 0.91	0.0289
Model 6	CRP flare-responder	0.25	0.12 to 0.50	0.0001
	HER-2 (positive)	0.76	0.32 to 1.59	0.4854

ECOG PS, Eastern Cooperative Oncology Group performance status; HER-2, human epidermal growth factor-2; chemo, chemotherapy; targeted, targeted therapy.

Model 1: include the group variable and the gastrectomy variable.

Model 2: include the group variable and the liver metastasis variable.

Model 3: include the group variable and the peritoneum metastasis variable.

Model 4: include the group variable and the combination therapy variable.

Model 5: include the group variable and the ECOG PS variable.

Model 6: include the group variable and the HER-2 variable.

In the multivariable analyses of OS and PFS, the impact of early CRP kinetics on survival outcomes was evaluated after adjusting for gastrectomy, liver metastasis, peritoneal metastasis, treatment regimen, ECOG PS, and HER-2 status. The results consistently demonstrated that, compared with CRP non-responders, CRP flare-responders had significantly lower risks of death and disease progression across all models (all p < 0.001) (**Tables 3**, **4**). Compared with CRP responders, CRP flare-responders showed a consistent trend toward lower risk of death across all models, although this did not reach statistical significance (all p > 0.05; all HRs < 1) (**Supplementary Table 2**). In contrast, the risk of disease progression was significantly reduced across all models (all p < 0.05) (**Supplementary Table 3**).

### Propensity score matching

Based on the results of the multivariable analysis, variables that showed a significant impact on survival outcomes were selected as matching covariates for pairwise 1:1 PSM. The matching variables included ECOG PS, HER-2, and treatment regimen. After matching, no significant differences in baseline characteristics were observed between the groups (**Supplementary Tables 4**–**6**).

Prior to the log-rank analyses of OS and PFS, Schoenfeld residual testing was performed on the matched cohort, confirming that the group variable did not violate the proportional hazards assumption, with all p-values exceeding 0.05 (**Supplementary Figure 3**).

Survival analysis demonstrated that CRP flare-responders had significantly longer median OS (29.36 months vs. 11.90 months, p < 0.001) and median PFS (19.60 months vs. 5.10 months, p < 0.001) than CRP non-responders. In addition, CRP flare-responders exhibited a significantly longer median PFS than CRP responders (17.80 months vs. 7.50 months, p = 0.014). However, the difference in median OS between CRP flare-responders and CRP responders did not reach statistical significance (26.80 months vs. 13.00 months, p = 0.172) (**Supplementary Table 7**).

### Sensitivity analysis

We conducted eight sensitivity analyses. In Model 1, all patients lost to follow-up were considered to have reached the study endpoint at the time of their last follow-up. In Models 2–4, patients with HER2-positive disease, an ECOG performance status of 2, and those who did not receive immunotherapy combined with chemotherapy were excluded, respectively. Model 5 excluded patients who died or experienced disease progression within 3 months, while Model 6 excluded those with death or progression within 6 months. In Model 7, patients were reclassified according to the original Fukuda criteria. In Model 8, patients were reclassified using only the pre-treatment CRP value before the first treatment as the baseline level. Across the survival analyses of all models, CRP flare-responders exhibited significantly lower risks of death and progression than non-CRP responders (all p < 0.05) (**Table 5**).

**Table 5:** Sensitivity analyses for overall survival and progression-free survival.

Model	Sample size (n)				Overall survival		
	CRP flare-responder	CRP responder	Non-CRP responder	HR (95%CI) for CRP flare-responder	P value	HR (95%CI) for CRP responder	P value
Model 1	21	18	20	0.29(0.14-0.57)	0.0004	0.58(0.29-1.13)	0.1109
Model 2	16	16	19	0.21(0.09-0.47)	0.0001	0.38(0.50-0.23)	0.0591
Model 3	18	15	16	0.27(0.11-0.62)	0.00198	0.71(0.32-1.54)	0.3865
Model 4	14	15	14	0.17(0.06-0.41)	0.00008	0.46(0.21-0.99)	0.0491
Model 5	21	18	19	0.27(0.13-0.57)	0.00058	0.56(0.27-1.15)	0.1146
Model 6	21	18	17	0.30(0.14-0.64)	0.00194	0.63(0.30-1.31)	0.21123
Model 7	22	23	14	0.26(0.12-0.61)	0.00223	0.53(0.25-1.14)	0.09966
Model 8	19	26	14	0.21(0.09-0.49)	0.000415	0.46(0.23-0.96)	0.03946
Model	Sample size (n)				Progression-free survival		
	CRP flare-responder	CRP responder	Non-CRP responder	HR (95%CI) for CRP flare-responder	P value	HR (95%CI) for CRP responder	P value
Model 1	21	18	20	0.27(0.14-0.53)	0.0001	0.62(0.32-1.18)	0.1456
Model 2	16	16	19	0.18(0.08-0.39)	0.00001	0.50(0.24-0.99)	0.0497
Model 3	18	15	16	0.27(0.12-0.58)	0.00095	0.72(0.34-1.50)	0.37143
Model 4	14	15	14	0.10(0.03-0.25)	0.000001	0.34(0.15-0.76)	0.00828
Model 5	21	17	17	0.28(0.13-0.61)	0.00127	0.56(0.26-1.17)	0.12273
Model 6	19	13	7	0.34(0.13-0.98)	0.04578	0.64(0.23-1.87)	0.39957
Model 7	22	23	14	0.26(0.12-0.56)	0.000735	0.64(0.33-1.30)	0.20749
Model 8	19	26	14	0.25(0.11-0.55)	0.000678	0.58(0.30-1.17)	0.12757

Model 1: All patients lost to follow-up were considered to have experienced death or disease progression.

Model 2: Exclude patients with positivity of HER-2.

Model 3: Exclude patients with an ECOG PS of 2.

Model 4: Patients treated with immunotherapy in combination with chemotherapy were included.

Model 5: Exclude patients who died or experienced disease progression within 3 months.

Model 6: Exclude patients who died or experienced disease progression within 6 months.

Model 7: Patients were reclassified according to the criteria proposed by Fukuda et al.

Model 8: Patients were reclassified using only the pre-treatment CRP level measured before the first treatment as the baseline value.

## Discussion

This retrospective study investigated the association between early CRP kinetics and clinical outcomes in patients with MSS mGC receiving PD-1 inhibitor therapy. We found that, compared with non-CRP responders, CRP flare-responders achieved a higher ORR and experienced significantly prolonged PFS and OS, with significantly reduced risks of disease progression and death. In addition, compared with CRP responders, CRP flare-responders showed no significant differences in ORR or risk of death; however, a significantly lower risk of disease progression was observed. These associations remained robust in multivariable analyses, PSM cohorts, and multiple sensitivity analyses, suggesting that early CRP kinetics may serve as a useful biomarker for monitoring treatment efficacy in this patient population.

Our findings are generally consistent with prior reports in other solid tumors, including renal cell carcinoma, non-small cell lung cancer, hepatocellular carcinoma and urothelial carcinoma, where CRP flare-response has been associated with improved clinical outcomes (12–15). Taken together, these findings suggest that transient inflammatory activation shortly after treatment initiation may represent a favorable biological response to immune checkpoint inhibition and may therefore provide clinically meaningful prognostic information.

PD-1 inhibitor-based therapy has become an important component of first-line treatment for mGC. However, treatment responses remain highly heterogeneous. Therefore, identifying reliable early biomarkers of treatment efficacy is of considerable clinical importance. Such biomarkers may facilitate timely treatment evaluation and help distinguish effective immune activation from atypical response patterns. This need is particularly relevant in MSS gastric cancer, in which the overall responsiveness to immunotherapy is generally lower and treatment benefit varies substantially among patients.

Previous studies have shown that baseline inflammatory biomarkers, such as the neutrophil-to-lymphocyte ratio and interleukin levels, are associated with survival outcomes in patients with advanced gastric cancer receiving immune checkpoint inhibitor therapy (16–18). Regarding CRP, elevated CRP levels after immunotherapy have been reported to be associated with hyperprogressive disease and poor survival outcomes, whereas higher baseline CRP levels may also predict an increased risk of mortality (19–21). Furthermore, CRP has been widely recognized as a prognostic biomarker in various malignancies (22, 23).

However, most previous investigations have relied on static measurements or short-term changes in inflammatory markers. Such approaches may not adequately capture the dynamic interplay among host immunity, systemic inflammation, and tumor evolution throughout treatment. In contrast, early CRP kinetics represent a continuous biological process and may therefore provide a more comprehensive assessment of treatment-induced immune activation than conventional inflammatory biomarkers.

To improve the clinical applicability of CRP kinetics, we modified the classification criteria originally proposed by Fukuda et al. (12) by incorporating the dual-threshold concept described by Wu et al. in cancer treatment models (24) and adapting the criteria to routine clinical practice. First, we set both the minimum threshold for CRP elevation and the baseline threshold for defining CRP responders at 10 mg/L. Since CRP levels exceeding 10 mg/L are generally regarded as indicative of clinically meaningful systemic inflammation, this threshold reduces the likelihood that minor biological fluctuations are misclassified as inflammatory responses. Second, we included the CRP level measured before the second treatment cycle as an additional baseline reference, given that CRP levels measured before the first treatment may be transiently elevated by mild postoperative pulmonary infection, anastomotic inflammation, or subclinical infection, thereby potentially compromising the accuracy of baseline CRP assessment. Finally, given that some patients received PD-1 inhibitors every three weeks, the time window for defining a CRP flare response was extended to 6 weeks to accommodate differences in treatment schedules. To address concerns that these modifications might influence patient classification, we conducted sensitivity analyses using both the original Fukuda criteria and the pre-first-treatment CRP value as the sole baseline reference. The survival advantages observed in CRP flare-responders remained consistent across all sensitivity analyses, supporting the robustness of our findings.

The underlying biological mechanisms linking CRP kinetics to immunotherapy efficacy remain incompletely understood. Increased expression of inflammatory mediators following immunotherapy is often accompanied by immune-related adverse events (4), and this elevation in inflammatory activity may reflect the activation of the host immune response. Therefore, we posit that the CRP flare-response reflects an acute inflammatory reaction associated with immune activation. Certain anticancer therapies, including oncolytic viruses, chemotherapeutic agents, targeted anticancer drugs, and physical interventions, can induce immunogenic cell death. This process triggers the release of damage-associated molecular patterns and various inflammatory mediators, leading to an acute and robust inflammatory response that promotes the infiltration of CD8^+^ T cells, CD4^+^ T cells, and natural killer cells into the tumor microenvironment (25–28). This process could transiently elevate CRP levels, followed by a decline as tumor burden decreases and immune homeostasis is restored. However, these hypotheses remain speculative and require further validation through mechanistic and translational studies.

Several limitations should be acknowledged. First, this was a single-center retrospective study with a relatively small sample size and a limited number of outcome events. Consequently, not all potential prognostic factors could be incorporated into multivariable analyses, which may have resulted in residual confounding. Second, although PSM and multiple sensitivity analyses yielded consistent results, unmeasured confounders inherent to retrospective studies cannot be completely excluded. Third, several potentially important biomarkers, including programmed death-ligand 1 expression and Claudin18.2 status, were not available for all patients and therefore could not be comprehensively evaluated. Finally, owing to the limited number of CRP flare-responders, further stratified analyses according to distinct CRP kinetic patterns were not feasible.

Future studies involving larger multicenter cohorts and prospective validation are required to confirm the predictive value of early CRP kinetics in MSS metastatic gastric cancer. In addition, accumulating evidence suggests that alterations in proteasomal deubiquitination, mutational landscapes, and mitochondrial metabolic activity may influence tumor progression, immune escape, and therapeutic resistance (29–31). Whether acute inflammatory responses induced during immunotherapy interact with these biological processes remains unclear. Elucidating these mechanisms may provide deeper insight into the relationship between systemic inflammation and antitumor immunity.

Despite these limitations, our findings suggest that early CRP kinetics represent a simple, inexpensive, and widely accessible biomarker that may assist in monitoring immune activation and predicting treatment outcomes in MSS mGC patients receiving PD-1 inhibitor-based therapy.

## Conclusion

Among patients with MSS mGC receiving first-line PD-1 inhibitor therapy, CRP flare response was associated with improved OR, PFS, and OS. Early CRP kinetics may represent a promising predictive biomarker for immunotherapy efficacy in MSS mGC. Further large-scale prospective studies are warranted to validate these findings.

## Data Availability

The original contributions presented in the study are included in the article/**Supplementary Material**. Further inquiries can be directed to the corresponding author.
