# Supplementary material for: C-reactive protein flare-response predicts the efficacy of PD-1 inhibitors in metastatic gastric cancer
Source: Front Immunol. 2026 Jul 1;17:1802864. doi: 10.3389/fimmu.2026.1802864 (PMC13368867; doi:10.3389/fimmu.2026.1802864)
Supplement: Supplementary file 1 [file DataSheet1.docx]

**Supplementary Figures**


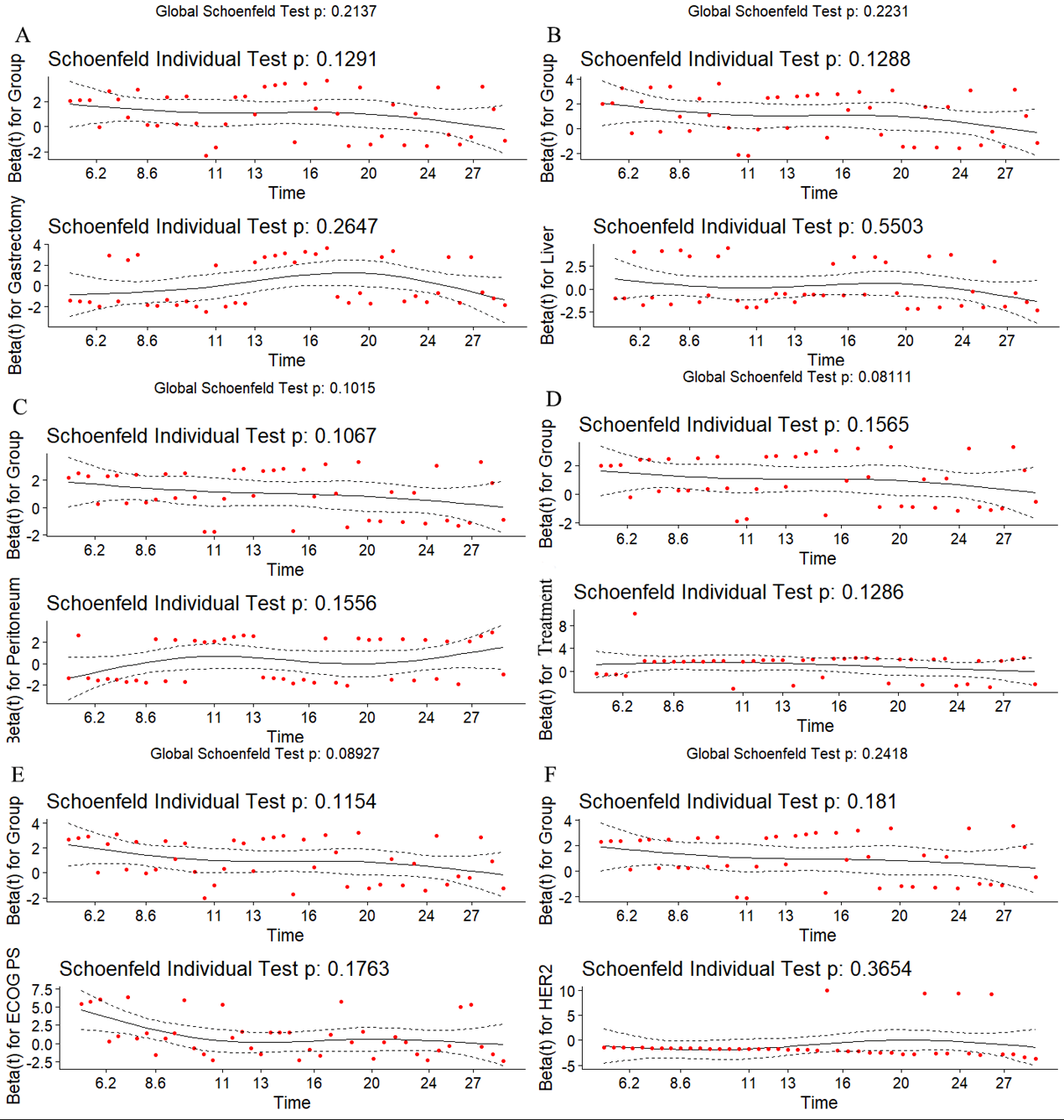


**Supplementary Figure 1.** Schoenfeld residual tests for the multivariable Cox regression model of overall survival. (A) group and gastrectomy (B) group and liver metastasis (C) group and peritoneum metastasis (D) group and combination therapy; (E) group and ECOG PS (F) group and HER-2


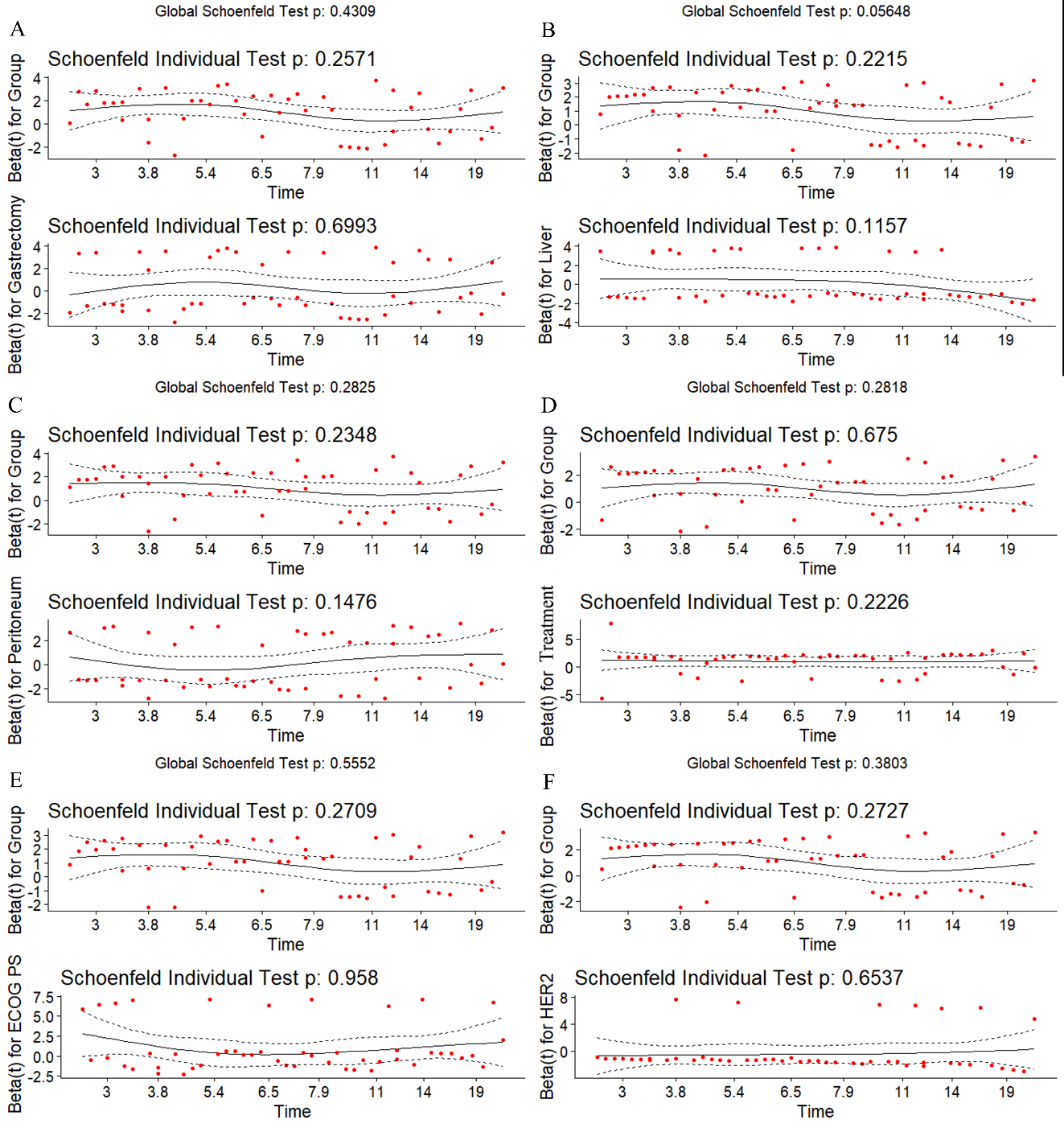


**Supplementary Figure 2.** Schoenfeld residual tests for the multivariable Cox regression model of progression-free survival. (A) group and gastrectomy (B) group and liver metastasis (C) group and peritoneum metastasis (D) group and combination therapy; (E) group and ECOG PS (F) group and HER-2


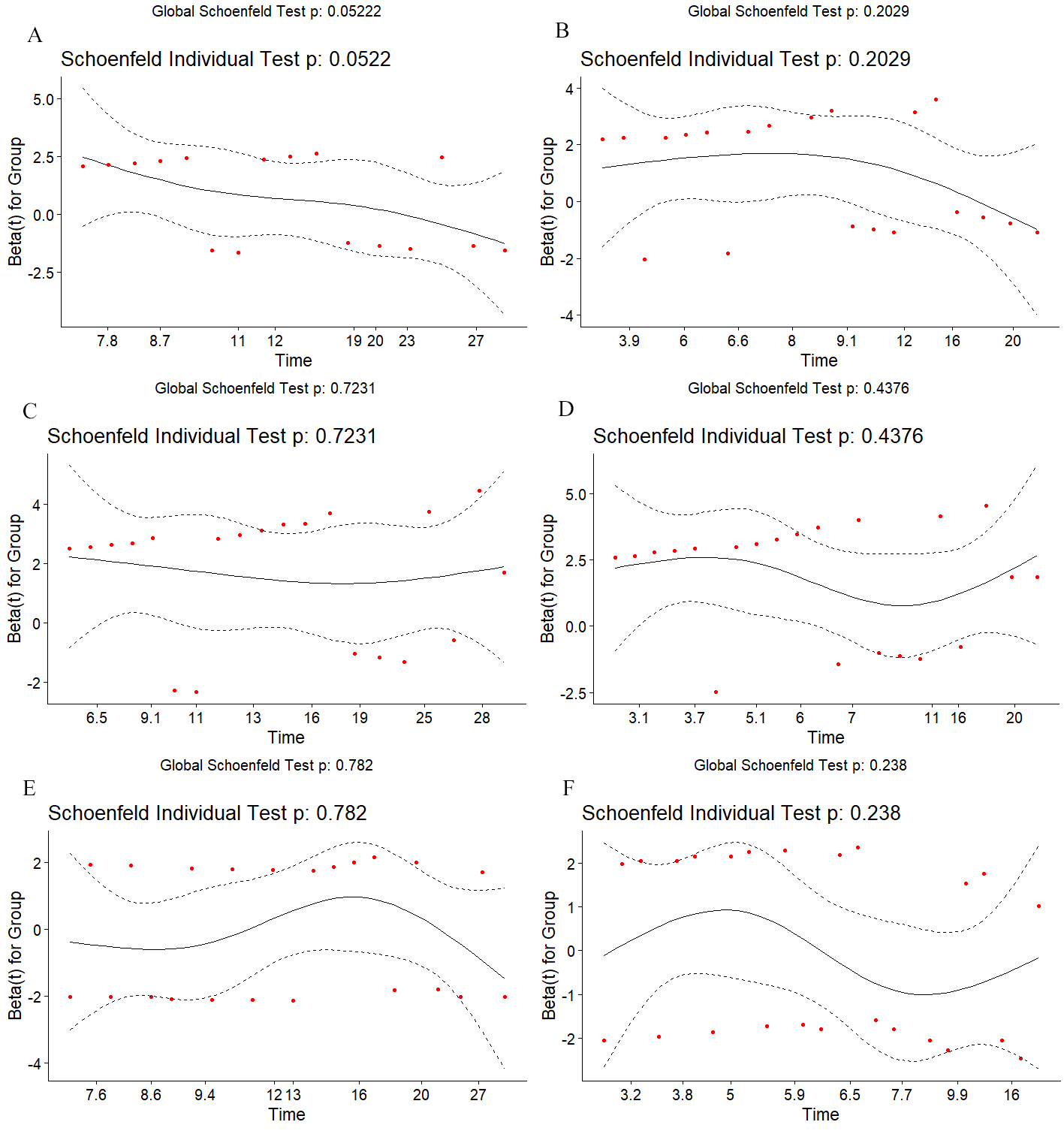


**Supplementary Figure 3**. Schoenfeld residual tests for overall survival and progression-free survival in the propensity score-matched cohort. (A) overall survival and (B) progression-free survival in CRP flare-responders and CRP responders; (C) overall survival and (D) progression-free survival in CRP flare-responders and non-CRP responders; (E) overall survival and (F) progression-free survival in CRP responders and non-CRP responders.

**Supplementary Tables**

**Supplementary Table 1**. Univariate and multivariate analysis for objective response

| Univariate analysis | | | | | |
| --- | --- | --- | --- | --- | --- |
|  | |  | OR | 95%CI | P value |
| Group | | CRP flare-responder | 3.08 | 0.67 to14.08 | 0.147 |
|  | | Non-CRP responder | 0.26 | 0.03 to 2.79 | 0.268 |
|  | | CRP responder | Reference | Reference | Reference |
| Multivariate analysis | | | | | |
|  | |  | OR | 95%CI | P value |
| Model 1 | CRP flare-responder | | 3.09 | 0.66 to 14.51 | 0.153 |
| Model 2 | CRP flare-responder | | 3.28 | 0.67 to 15.99 | 0.141 |
| Model 3 | CRP flare-responder | | 3.99 | 1.79 to 19.97 | 0.093 |
| Model 4 | CRP flare-responder | | 3.10 | 0.65 to 14.81 | 0.155 |

Model 1: include the group variable and the gastrectomy variable.

Model 2: include the group variable and the liver metastasis variable.

Model 3: include the group variable and the peritoneum metastasis variable.

Model 4: include the group variable and the combination therapy variable.

**Supplementary Table 2**. Univariate and multivariate analysis for overall survival.

| Univariate analysis | | | | | |
| --- | --- | --- | --- | --- | --- |
|  | | Variable | HR | 95%CI | P value |
| Group | | CRP flare-responder | 0.49 | 0.22 to 1.07 | 0.073 |
|  | | Non-CRP responder | 1.88 | 0.92 to 3.85 | 0.086 |
|  | | CRP responder | Reference | Reference | Reference |
| Multivariate analysis | | | | | |
|  | Variable | | HR | 95%CI | P value |
| Model 1 | CRP flare-responder | | 0.48 | 0.22 to 1.06 | 0.070 |
| Model 2 | CRP flare-responder | | 0.53 | 0.23 to 1.21 | 0.128 |
| Model 3 | CRP flare-responder | | 0.47 | 0.21 to 1.04 | 0.064 |
| Model 4 | CRP flare-responder | | 0.49 | 0.22 to 1.10 | 0.086 |
| Model 5 | CRP flare-responder | | 0.49 | 0.22 to 1.12 | 0.090 |
| Model 6 | CRP flare-responder | | 0.50 | 0.23 to 1.10 | 0.084 |

Model 1: include the group variable and the gastrectomy variable.

Model 2: include the group variable and the liver metastasis variable.

Model 3: include the group variable and the peritoneum metastasis variable.

Model 4: include the group variable and the combination therapy variable.

Model 5: include the group variable and the ECOG PS variable.

Model 6: include the group variable and the HER-2 variable.

**Supplementary Table 3**. Univariate and multivariate analysis for progression-free survival.

| Univariate analysis | | | | | |
| --- | --- | --- | --- | --- | --- |
|  | | Variable | HR | 95%CI | P value |
| Group | | CRP flare-responder | 0.40 | 0.19 to 0.83 | 0.014 |
|  | | Non-CRP responder | 1.61 | 0.82 to 3.15 | 0.164 |
|  | | CRP responder | Reference | Reference | Reference |
| Multivariate analysis | | | | | |
|  | Variable | | HR | 95%CI | P value |
| Model 1 | CRP flare-responder | | 0.37 | 0.17 to 0.79 | 0.010 |
| Model 2 | CRP flare-responder | | 0.41 | 0.19 to 0.87 | 0.021 |
| Model 3 | CRP flare-responder | | 0.38 | 0.18 to 0.81 | 0.012 |
| Model 4 | CRP flare-responder | | 0.38 | 0.12 to 0.81 | 0.012 |
| Model 5 | CRP flare-responder | | 0.39 | 0.18 to 0.83 | 0.015 |
| Model 6 | CRP flare-responder | | 0.41 | 0.20 to 0.85 | 0.016 |

Model 1: include the group variable and the gastrectomy variable.

Model 2: include the group variable and the liver metastasis variable.

Model 3: include the group variable and the peritoneum metastasis variable.

Model 4: include the group variable and the combination therapy variable.

Model 5: include the group variable and the ECOG PS variable.

Model 6: include the group variable and the HER-2 variable.

**Supplementary Table 4**. Baseline characteristics of CRP flare-responder and CRP responder after propensity score matching.

|  |  | Total cohort, n (%) | CRP flare-responder, n (%) | CRP responder, n (%) | P value |
| --- | --- | --- | --- | --- | --- |
| Variables |  |  |  |  |  |
| No. of patients |  | 26 | 13(50) | 13(50) |  |
| Age (years) | Median (IQR) | 63(58-66) | 64(59-67) | 63(58-67) | 0.479 |
| Gender | Male | 14(54) | 9(69) | 5(39) | 0.238 |
|  | Female | 12(46) | 4(31) | 8(62) |  |
| ECOG PS | 0 | 11(43) | 5(39) | 6(46) | 0.777 |
|  | 1 | 10(39) | 6(46) | 4(31) |  |
|  | 2 | 5(19) | 2(15) | 3(23) |  |
| Differentiation | Poor | 24(92) | 12(92) | 12(92) | 1.000 |
|  | Moderate | 2(8) | 1(8) | 1(8) |  |
| HER-2 | Positive | 2(8) | 1(8) | 1(8) | 1.000 |
|  | Negative | 24(92) | 12(92) | 12(92) |  |
| Gastrectomy | Yes | 7(27) | 4(31) | 3(23) | 1.000 |
|  | No | 19(73) | 9(69) | 10(77) |  |
| Sites of metastases | Lung | 4(15) | 1(8) | 3(23) | 0.593 |
|  | Bone | 3(12) | 0(0) | 3(23) | 0.220 |
|  | Peritoneum | 13(50) | 8(62) | 5(39) | 0.434 |
|  | lymph node | 3(12) | 2(15) | 1(8) | 1.000 |
|  | Liver | 9(65) | 3(23) | 6(46) | 0.411 |
|  | Ovary | 1(4) | 0(0) | 1(8) | 1.000 |
|  | Kidney | 1(4) | 1(8) | 0(0) | 1.000 |
| Combination therapy | Chemo | 22(85) | 11(85) | 11(85) | 1.000 |
|  | Chemo + targeted | 4(15) | 2 (15) | 2 (15) |  |
| CRP level before the first treatment (mg/L) | Median (IQR) | 11.4(6.1-24.8) | 6.1(4.1-24.7) | 23.9  (9.7-27.7) | 0.153 |

ECOG PS, Eastern Cooperative Oncology Group performance status; HER-2, human epidermal growth factor-2; chemo, chemotherapy; targeted, targeted therapy.

|  |  | Total cohort, n (%) | CRP flare-responder, n (%) | Non-CRP responder, n (%) | P value |
| --- | --- | --- | --- | --- | --- |
| Variables |  |  |  |  |  |
| No. of patients |  | 28 | 14(50) | 14(50) |  |
| Age (years) | Median (IQR) | 61(55-68) | 65(56-67) | 58(50-72) | 0.661 |
| Gender | Male | 19(65) | 10(71) | 9(64) | 1.000 |
|  | Female | 9(35) | 4(29) | 5(36) |  |
| ECOG PS | 0 | 11(39) | 6(43) | 5(36) | 1.000 |
|  | 1 | 12(43) | 6(43) | 6(43) |  |
|  | 2 | 5(18) | 2(14) | 3(21) |  |
| Differentiation | Poor | 26(93) | 13(93) | 13(93) | 1.000 |
|  | Moderate | 2(7) | 1(7) | 1(7) |  |
| HER-2 | Positive | 28(100) | 14(50) | 14(50) | / |
|  | Negative | 0(0) | 0(0) | 0(0) |  |
| Gastrectomy | Yes | 17(61) | 5(36) | 6(43) | 1.000 |
|  | No | 11(39) | 9(64) | 8(57) |  |
| Sites of metastases | Lung | 4(14) | 1(7) | 3(21) | 0.596 |
|  | Bone | 2(7) | 0(0) | 2(14) | 0.481 |
|  | Peritoneum | 12(43) | 8(57) | 4(29) | 0.252 |
|  | lymph node | 5(18) | 3(21) | 2(14) | 1.000 |
|  | Liver | 6(21) | 3(21) | 3(21) | 1.000 |
|  | Ovary | 3(11) | 0(0) | 3(21) | 0.222 |
|  | Kidney | 1(4) | 1(7) | 0(0) | 1.000 |
| Combination therapy | No | 2(7) | 1(7) | 1(7) |  |
|  | Chemo | 23(82) | 11(79) | 12(86) | 1.000 |
|  | Chemo + targeted | 3(11) | 2(14) | 1(7) |  |
| CRP level before the first treatment (mg/L) | Median (IQR) | 6.1(3.1-10.5) | 6.8(5.0-24.7) | 5.2  (2.5-8.9) | 0.528 |

**Supplementary Table 5**. Baseline characteristics of CRP flare-responder and non-CRP responder after propensity score matching.

ECOG PS, Eastern Cooperative Oncology Group performance status; HER-2, human epidermal growth factor-2; chemo, chemotherapy; targeted, targeted therapy.

|  |  | Total cohort, n (%) | CRP responder, n (%) | Non-CRP responder, n (%) | P value |
| --- | --- | --- | --- | --- | --- |
| Variables |  |  |  |  |  |
| No. of patients |  | 28 | 14(50) | 14(50) |  |
| Age (years) | Median (IQR) | 63(57-67) | 63(58-67) | 62(54-66) | 0.645 |
| Gender | Male | 16(57) | 7(50) | 9(64) | 0.704 |
|  | Female | 12(43) | 7(50) | 5(36) |  |
| ECOG PS | 0 | 10(36) | 6(43) | 4(29) | 0.394 |
|  | 1 | 14(50) | 5(36) | 9(64) |  |
|  | 2 | 4(14) | 3(21) | 1(7) |  |
| Differentiation | Poor | 25(89) | 12(86) | 13(93) | 1.000 |
|  | Moderate | 3(11) | 2(14) | 1(7) |  |
| Gastrectomy | Yes | 9(32) | 4(29) | 5(36) | 1.000 |
|  | No | 19(68) | 10(71) | 9(64) |  |
| Sites of metastases | Lung | 5(18) | 2(14) | 3(21) | 1.000 |
|  | Bone | 5(18) | 3(21) | 2(14) | 1.000 |
|  | Peritoneum | 10(36) | 5(36) | 5(36) | 1.000 |
|  | lymph node | 4(14) | 1(7) | 3(21) | 0.596 |
|  | Liver | 10(36) | 6(43) | 4(29) | 0.695 |
|  | Ovary | 3(11) | 1(7) | 2(14) | 1.000 |
|  | Kidney | 0(0) | 0(0) | 0(0) | / |
| Combination therapy | Chemo | 22(79) | 11(79) | 11(79) | 1.000 |
|  | Chemo + targeted | 6(21) | 3(21) | 3(21) |  |
| CRP level before the first treatment (mg/L) | Median (IQR) | 10.6(6.4-24.4) | 15.6(8.9-24.8) | 6.5  (1.8-16.4) | 0.360 |

**Supplementary Table 6.** Baseline characteristics of CRP responder and non-CRP responder after propensity score matching.

ECOG PS, Eastern Cooperative Oncology Group performance status; HER-2, human epidermal growth factor-2; chemo, chemotherapy; targeted, targeted therapy.

**Supplementary Table 7**. Overall survival and progression-free survival after propensity score matching.

| Model | Sample size (n) | | | Median overall survival time (month) | | | |
| --- | --- | --- | --- | --- | --- | --- | --- |
|  | CRP flare-responder | CRP responder | Non-CRP responder | CRP flare-responder | CRP responder | Non-CRP responder | P value |
| Model 1 | 13 | 13 | / | 26.80 | 13.00 | / | 0.172 |
| Model 2 | 14 | / | 14 | 29.36 | / | 11.90 | 0.000332 |
| Model 3 | / | 14 | 14 | / | 13.00 | 13.66 | 0.892 |
| Model | Sample size (n) | | | Median progression-free survival time (month) | | | |
|  | CRP flare-responder | CRP responder | Non-CRP responder | CRP flare-responder | CRP responder | Non-CRP responder | P value |
| Model 1 | 13 | 13 | / | 17.80 | 7.50 | / | 0.014 |
| Model 2 | 14 | / | 14 | 19.60 | / | 5.10 | 0.000070 |
| Model 3 | / | 14 | 14 | / | 6.70 | 5.66 | 0.907 |

Model 1: CRP flare-responder and CRP responder.

Model 2: CRP flare-responder and non-CRP responder.

Model 3: CRP responder and non-CRP responder.
